# Botulinum Toxin for Neuropathic Pain: A Review of the Literature

**DOI:** 10.3390/toxins7083127

**Published:** 2015-08-14

**Authors:** Hyun-Mi Oh, Myung Eun Chung

**Affiliations:** 1 Department of Rehabilitation Medicine, Seoul St. Mary’s Hospital, College of Medicine, the Catholic University of Korea, 222 Banpo-daero, Seocho-gu, Seoul 06591, Korea; E-Mail: hazel@cmcnu.or.kr; 2 Department of Rehabilitation Medicine, St. Paul’s Hospital, College of Medicine, the Catholic University of Korea, Wangsan-ro 180, Dongdaemoon-Gu, Seoul 02559, Korea

**Keywords:** botulinum toxins, BoNT/A, neuropathic pain, neuralgia, antinociceptive

## Abstract

Botulinum neurotoxin (BoNT), derived from *Clostridium botulinum*, has been used therapeutically for focal dystonia, spasticity, and chronic migraine. Its spectrum as a potential treatment for neuropathic pain has grown. Recent opinions on the mechanism behind the antinociceptive effects of BoNT suggest that it inhibits the release of peripheral neurotransmitters and inflammatory mediators from sensory nerves. There is some evidence showing the axonal transport of BoNT, but it remains controversial. The aim of this review is to summarize the experimental and clinical evidence of the antinociceptive effects, mechanisms, and therapeutic applications of BoNT for neuropathic pain conditions, including postherpetic neuralgia, complex regional pain syndrome, and trigeminal neuralgia. The PubMed and OvidSP databases were searched from 1966 to May 2015. We assessed levels of evidence according to the American Academy of Neurology guidelines. Recent studies have suggested that BoNT injection is an effective treatment for postherpetic neuralgia and is likely efficient for trigeminal neuralgia and post-traumatic neuralgia. BoNT could also be effective as a treatment for diabetic neuropathy. It has not been proven to be an effective treatment for occipital neuralgia or complex regional pain syndrome.

## 1. Introduction

Botulinum neurotoxin (BoNT), derived from *Clostridium botulinum*, has been used worldwide for not only cosmetic therapeutic purposes but also for the treatment of neurologic disorders, such as dystonia or spasticity [1,2]. It has been approved by the Food and Drug Administration (FDA) as a treatment for strabismus, blepharospasm, hemifacial spasm, focal dystonia, and upper limb spasticity in the United States [3].

BoNT has seven antigenically different serotypes (A–G) [4], and its main mechanism is the inhibition of acetylcholine (Ach) neurotransmitter release at presynaptic nerve terminals, which results in a reduction of muscle fiber activity [2,4,5]. BoNT inhibits the exocytosis of Ach from cholinergic nerve endings by endocytosing into the presynaptic membranes of neuromuscular junctions and cleaving proteins that are essential for Ach exocytosis [6]. These proteins are required for the docking of Ach-containing vesicles to presynaptic membranes. Without this docking, Ach cannot be released into the synaptic cleft, and the innervated structure can become paralyzed.

Each serotype of BoNT acts as a protease that specifically targets soluble *N*-ethylmaleimide-sensitive factor attachment protein receptor (SNARE) proteins by attaching a light chain, which is an active part of the toxin [7]. With the plasma membrane, the SNARE proteins form a core that is required to mediate the fusion of synaptic vesicles (SVs). The SNARE proteins include synaptosomal-associated protein of 25 kDa (SNAP-25), target membrane proteins, syntaxin, and the vesicle-associated membrane protein (VAMP)/synaptobrevin. BoNT/A—the most well-known toxin—and BoNT/E cleave SNAP-25 at two different sites. BoNT/B, /D, /F, and /G cleave VAMP/synaptobrevin at different sites, whereas BoNT/C cleaves both syntaxin and SNAP-25 [4,7].

Aside from blocking the release of Ach, it has been suggested that BoNT, particularly BoNT/A, may inhibit the release of local nociceptive neuropeptides, such as substance P, calcitonin gene-related peptide (CGRP), and glutamate, as well as the expression of the transient receptor potential vanilloid 1 (TRPV1) [8,9,10,11]. Through this process, BoNT/A may inhibit neurogenic inflammation and peripheral sensitization. In discovering the different mechanisms of the different BoNTs, *in vivo* data have been generated that suggest that BoNT can serve as a potential treatment for pain. In addition, the FDA approved Botox® for the treatment of chronic migraines in 2010 after investigating its efficacy in reducing the frequency and intensity of chronic migraines [12,13]. BoNT/A is the only approved prophylactic drug, with a peripheral application [14]. In addition to the impairment of SNARE-mediated synaptic vesicle fusion to nerve terminals, this study demonstrated that BoNT/A selectively inhibits *C*- but not A_δ_-menigeal nociceptors. BoNT inhibited mechanical transduction in suture branches of meningeal nociceptors during extracranial application. Burstein *et al.* [14] suggest that during the prophylactic application of BoNT/A for migraines, it prevents high-threshold mechanosensitive ion channels from fusing into the nerve terminal membrane and lowers the neuronal surface expression of these channels.

The spectrum of the usage of BoNT/A for the treatment of pain is increasing, and the possibility that BoNT/A can be used as a treatment for a wider variety of pain disorders is currently being explored. However, the entire process by which BoNT/A exerts pain relief is not yet clear.

Neuropathic pain is a type of pain that is “caused by a lesion or disease of the somatosensory system” [15], and recent studies have suggested that BoNT/A is effective for the treatment of different clinical conditions that present with neuropathic pain, such as postherpetic neuralgia, diabetic neuropathy, post-traumatic neuralgia, complex regional pain syndrome, trigeminal neuralgia, and occipital neuralgia [12,16,17,18].

The aim of this review is to investigate the mechanisms behind the antinociceptive actions of BoNT/A, its axonal transport, and its anti-inflammatory properties and to provide a compilation of data that report on the therapeutic use of BoNT injections for neuropathic pain conditions by assigning levels of evidence according to the American Academy of Neurology guidelines. A literature search was conducted using the PubMed and OvidSP databases (1966–May 2015) with the following cross-searching terms: “Botulinum toxins, type A”, “botulinum toxin”, “botulinum neurotoxin”, “neuralgia”, “neuropathy”, “pain”, “chronic pain”, and “neuropathic pain”. The results included randomized controlled trials, observational studies, and case reports. Additional references were found by reviewing individual references from the articles, which were searched using PubMed.

## 2. Mechanisms of the Antinociceptive Effects of Botulinum Toxin

There have been several preclinical studies showing that BoNT/A inhibits the release of neurotransmitters that regulate pain and inflammation [11,19]. A rat formalin-induced inflammatory pain model was used in an early *in vivo* preclinical study that showed the antinociceptive effects of BoNT/A [11]. A subcutaneous BoNT/A injection reduced inflammatory hyperalgesia and edema. An intrathecal injection of BoNT/A showed similar analgesic effects in animal models of formalin-induced pain [19].

Welch *et al.* observed that BoNT inhibits the release of substance P from cultured embryonic rat dorsal root ganglia neurons [20]. In this study, dorsal root ganglia neurons showed different sensitivities for each serotype of BoNT; they were the most sensitive to BoNT/A, suggesting differences in receptor affinity between the various BoNT serotypes. The effects of BoNT/A cleavage of SNAP 25 appeared after two hours, whereas substance P secretion was inhibited after four hours. This effect of BoNT/A lasted for up to 15 days. In rat trigeminal ganglia cells, BoNT/A directly diminished the amount of calcitonin gene-related peptide (CGRP), an inflammatory neuropeptide [21]. Incubating the cell cultures with BoNT/A was more effective in suppressing stimulated CGRP secretion than incubating them with a control. Substance P and CGRP are neurotransmitters produced by dorsal root ganglia neurons, mainly C fibers, a primary nociceptive afferent. This suggests that BoNT/A inhibits the release of these neurotransmitters from primary sensory neurons [8]. These neurotransmitters can act on blood vessels and induce vasodilatation. This can cause flares of weals to appear on the skin around lesions. By inhibiting the release of these neurotransmitters, BoNT/A can decrease tenderness and pain in areas where sensitized nociceptors are pathologically altered. Ultimately, substance P and CGRP contribute to peripheral sensitization during inflammation and indirectly reduce central sensitization [6,8,22].

**Figure 1:**
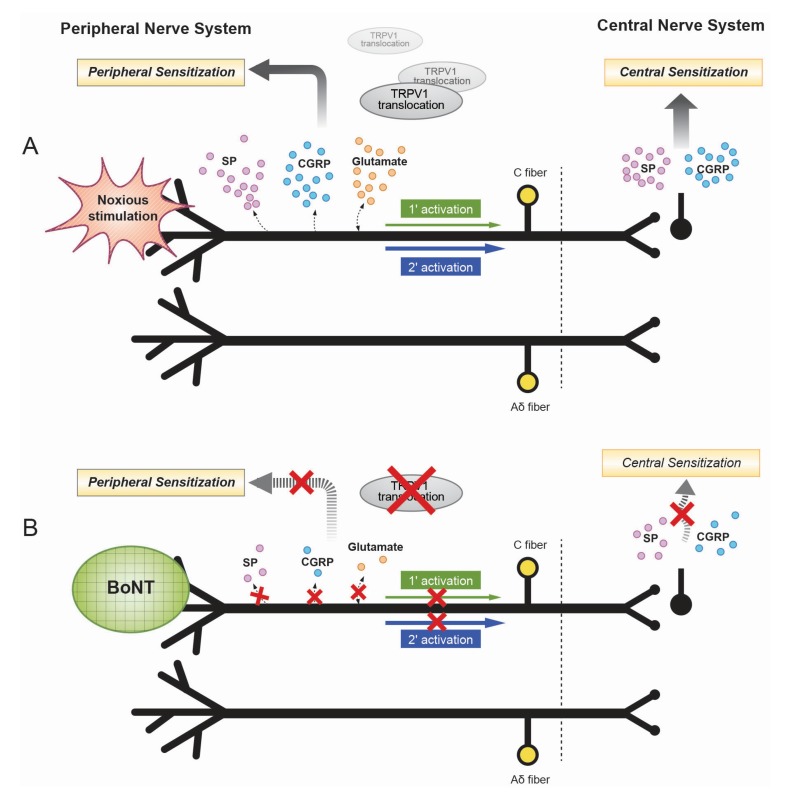
(**A**) Illustrated mechanism of peripheral and central nerve system sensitization. Noxious stimulation may lead to peripheral sensitization through release of neuropeptides and inflammatory mediators. The peripheral sensitization may result in sensitization of the central nerve system. SP indicates substance P; CGRP, calcitonin gene-related peptide; TRPV1, transient receptor potential vanilloid 1. (**B**) The antinociceptive mechanism of botulinum neurotoxin (BoNT) in the treatment of neuropathic pain including decrease in peripheral SP, CGRP, glutamate, TRPV1 receptor translocation, leading to direct block of peripheral sensitization. As substance P and CGRP secretion are blocked within central nerve system, central sensitization is also indirectly reduced.

In addition, BoNT can decrease the delivery of the transient receptor potential vanilloid 1 (TRPV1) to neuron cell membranes [23]. TRPV1 is an ionotropic receptor that intensifies the excitability of nociceptors, responding to noxious stimuli, such as heat and proalgesic substances. BoNT/A can inhibit the SNARE-mediated translocation of TRPV1 to the plasma membrane [24]. TRPV1 has an important role during the processing of peripheral thermal and inflammatory pain. Proalgesic agents can up-regulate TRPV1 expression and channel activity [23]. BoNT/A is also effective in inhibiting the release of excitatory neurotransmitters, such as glutamate. In a study that utilized a guinea pig formalin-induced pain model [25,26], glutamate concentrations decreased after an injection of BoNT compared with an injection of saline. Glutamate secretion was also inhibited in a synaptosome preparation from the cerebral cortex of a guinea pig (Figure 1).

Paterson *et al.* found that BoNT/A selectively increased the threshold of mechanical pain without changing the thermal detection or pain threshold [27]. The effect of BoNT/A on mechanotransfution was evaluated at the cellular level. Cultured mouse dorsal root ganglion neurons were exposed to BoNT/A. BoNT treatment showed no change in rapidly and intermediately adapting, mechanically activated inward currents, while there was a significant decrease in the proportion of cells expressing a slowly adapting, mechanically gated current with local pressure to the cell soma. There was no effect of BoNT/A on the activation threshold of mechanically gated currents with a slight decrease in the peak current intensity. It has been believed that the slowly adapting, mechanically gated current in dorsal root ganglion neurons has been linked to noxious mechanosensation; conotoxin noxious mechanosensation blocker-1 selectively inhibits slowly adapting, mechanically activated currents. This action increases mechanical pain threshold without changing light touch or heat sensitivity [28]. The responsible channels for this current are not known yet. More possible explanation can be effects of BoNT/A on the trafficking of mechanosensitive channels. BoNT would block vesicular traffic and suppress protein kinase C activation. Mechanically gated currents could be upregulated by inflammatory mediators in sensory neuron cultures [27,29].

BoNT/A has been used as a treatment for pain associated with neuromuscular hyperactivity disorders, such as spasmodic torticollis and cervical dystonia, for decades [3,30]. It was initially believed that BoNT/A reduced pain by inducing muscular relaxation through inhibiting the release of acetylcholine [6]; this might indirectly reduce the painful ischemia that can be caused by a hyper-contracted muscle. However, there have been a few cases in which pain relief was observed even in patients who did not show any improvements in hyperactive muscle contraction [31]. Additionally, clinicians have reported cases of pain relief occurring before muscle relaxation [32]. It was also discovered that pain relief existed even after the effects of muscle relaxation disappeared, measured by reduced bite force in patients treated for temporomandibular disorders [33]. In a study by Relja and Klepac [34], pain relief was observed one week after BoNT/A injection in patients with spasmodic torticollis, whereas paralytic effects started two weeks after the injection. Lower doses (50 units) of BoNT/A were necessary to induce analgesic effects, whereas 100 to 150 units were needed to create a paralytic effect [34]. This implies that the mechanism behind the antinociceptive effects of BoNT/A is more complex than muscle relaxation and that these effects on pain can be explained.

There are efforts to find additional explanations for BoNT as a treatment. Xiao *et al.* were interested in the potential interaction between BoNT/A and transmembrane channels/receptors, which are closely linked to pain [35]. They performed a study to determine the effects of BoNT/A on neuropathic pain and the expression of purinergic P2X_3_ receptor in the dorsal root ganglion of rats with neuropathic pain caused by L5 ventral root transaction. Either saline or BoNT/A was injected to plantar surfaces. Before and after the operation, behavioral tests were done. After 3, 7, 14 and 21 days, immunoreactivity was used to detect the expression of P2X_3_ receptors in the dorsal root ganglion. Nerve transaction induced bilateral mechanical allodynia and over-expression of P2X_3_ receptors in the dorsal root ganglion of the rats. Subcutaneous intraplantar injection of BoNT/A reduced the neuropathic behavior and the over-expressed P2X_3_ receptors of nociceptive neurons. This study shows over-expressed P2X_3_ receptors in this ventral root transaction model and reveals a novel mechanism of BoNT, acting on nociceptive neurons.

For phantom limb pain and phantom sensation, in cases where the nerves are transected, the pain is characterized by the cortical perception of an amputated body part [36]. There are suggested pathophysiologies, including ectopic activities at the neuroma, which are formed at the transected nerve ending in the residual limb. Early studies of rat transected sciatic nerves showed that the site of neuroma is complicated and dynamic. Images of neuromas show multiple sprouts growing out from each cut axon and can travel in many directions, including back along the axon. The neuroma can increase sympathetic tone through central plasticity and sensitization. There are usually immediate changes of chemical transport including the dorsal root ganglion and from peripheral to central nervous system. The central pathways involve hyperexcitability in the spinal cord, and this can cause somatosensory cortical reorganization.

To increase the potential of BoNT for therapeutic usage, developing a recombination of BoNT can be promising. Before discussing the recombination, the structure of BoNT/A complex should be discussed first. As mentioned, there are the seven distinct serotypes (A–G) which selectively block different proteins of the SNARE. While toxin types C and D are causes of illness in animals, the toxin types A, B, E, F are well-known causes of human botulism [4,37]. The serotypes cleave proteins, required for the synaptic vesicles to fuse to the presynaptic membrane and block the exocytosis of neurotransmitters. The BoNT consist of a light chain which is linked to a heavy chain through a disulphide bridge [38,39,40]. The heavy chain has a carboxy terminal membrane acceptor-binding domain, and BoNT/A can bind to overactive nerve endings with high affinity through the *C*-terminal half of the heavy chain. The heavy chain also has a translocation domain at the *N*-terminal which can mediate the toxin binding to nerve terminals. The light chain enters the presynaptic cytosol and prevents neurotransmitter release by the enzymatic cleavage of SNAP-25 [41].

Cleaving SNAP-25 at different sites, BoNT/A and BoNT/E have very different durations of actions [42]. The paralytic effects of BoNT/A last for months in humans and for weeks in mice; BoNT/E acts for short periods, in the span of days. While BoNT/A reduces the fusion rate, BoNT/E completely inhibits vesicle fusion, akin to the effects of VAMP [41]. In addition, when BoNT/E inhibits neurotransmitter release, it is not influenced by treatments that elevate cytosol (Ca^2+^). BoNT/E can enter the cultured neuron faster than BoNT/A. With higher potency, it can inhibit transmission at the neuromuscular junction more quickly. These properties of BoNT/E are attractive to improve therapeutic versions of BoNT; however, BoNT/E also has a downside—it can produce transient muscle weakness—compared with the long action duration of BoNT/A. To find a responsible domain for the properties, the *C*-terminal half of the heavy chain was exchanged to create chimeras.

In Wang *et al.*’s study, chimeras were produced by swapping the *C*-terminal half of the heavy chain domains between BoNT/A and /E [39]. Recombinant EA has the light chain and *N*-terminal half of the heavy chain domains of BoNT/E and the *C*-terminal half of the heavy chain of BoNT/A, while AE comprises the light chain and *N*-terminal half of the heavy chain of BoNT/A. Swapping BoNT domains yields novel features; chimera EA was fast-acting and more potent than BoNT/A at the neuromuscular junction. EA was as potent and fast as BoNT/E, and they were equivalently sensitive to dose-dependent inhibition of the vesicular proton pump. AE produced persistent muscle weakness, which can be therapeutic potential. This advanced technology can bring further advances to the therapeutic use of BoNT.

## 3. Axonal Transport of Botulinum Toxin

Antonucci *et al.* provided biochemical evidence that BoNT/A can cleave SNAP-25 distant from an injection site, suggesting that axonal transport of BoNT/A occurs within central neurons [43]. There have been reports on the bilateral effects of BoNT/A after a unilateral injection [44,45,46]. In a study by Bach-Rojecky *et al.*, BoNT/A injections were administered to a rat model of diabetic neuropathy that was induced by streptozotocin [45]. After BoNT/A application, mechanical and therapy hyperalgesia improved on both the ipsilateral and contralateral sides. This antinociceptive effect lasted for more than 15 days.

In another study, Bach-Rojecky *et al.* suggested that the central antinociceptive effects of BoNT/A are driven by axonal transport, which was demonstrated in a unilateral acidic saline-induced animal model with bilateral hyperalgesia [44]. This “mirror pain”, induced by unilateral injections of acidic saline, was suggested to be of central origin. Therefore, the authors had expected that applying BoNT/A peripherally would not show any effects on bilateral pain. However, bilateral pain was reduced when a unilateral subcutaneous injection of BoNT/A (5 units/kg) was performed on the ipsilateral side. In addition, BoNT/A (0.5 units/kg) still reduced bilateral pain when it was injected into a proximal region of a distally transected sciatic nerve. In both cases, a unilateral injection reduced acidic saline-induced bilateral mechanical hyperalgesia.

In an animal study, the antinociceptive effects of BoNT/A were investigated on paclitaxel-induced peripheral neuropathy [46]. After being injected with paclitaxel, a chemotherapeutic drug, rats showed a decreased withdrawal nociceptive reflex bilaterally in their hind paws. Again, after the toxin was injected on the unilateral side, analgesic effects were observed bilaterally. These data suggest that BoNT/A might affect the central nervous system (CNS) [38], as they cannot be explained solely by BoNT/A’s actions on peripheral nerve endings. The toxin might have diffused hematogenously, but the administered dose (20 units/kg subplantar injection) was not sufficiently high to induce systemic side effects. Furthermore, the protein is not sufficiently small to pass through the blood-brain barrier. Therefore, one possible theory is that BoNT/A is axonally transported from the periphery to the CNS. However, this theory has not yet been proven [38].

In the previously mentioned study by Bach-Rojecky *et al.*, to prevent retrograde axonal transport, an axonal transport blocker, colchicine, was injected into the ipsilateral sciatic nerve, after which the bilateral antinociceptive effects disappeared [44]. An injection of colchicine into the contralateral side did not eliminate the antinociceptive effects, suggesting that the axonal transport of BoNT/A from peripheral nerves toward the CNS.

In a study by Wiegand *et al.*, ^125^I-BoTN/A was injected unilaterally into the gastrocnemius muscle of a cat to trace the toxin [47]. After the injection, radioactivity was progressively observed first in the sciatic nerve and then in the ipsilateral spinal ventral roots and the ipsilateral spinal cord segment after 48 h. This study suggested the possibility of the toxin moving into the CNS via axonal transport; however, it could not prove that the enzymatically active toxin reached the CNS.

There have been studies that have shown the opposite effect. In a study by Tang-Liu *et al.*, an ^125^I-radiolabeled BoNT-hemagglutinin complex (BoNT/A-complex) and ^125^I-radiolabeled free BoNT/A were compared; these radioiodinated compounds were injected into the gastrocnemius muscles of cats and the eyelids of rabbits and measured at different time-points [48]. No systemic effects were observed in either the rats or rabbits. The ^125^I-radiolabeled BoNT/A-complex and free BoNT/A remained near the injection sites. Radioactivity was detected in distal tissues, such as the thyroid gland, and contralateral muscles and skin; however, the authors suggested that this was mainly due to the low molecular weights of ^125^I-iodide and ^125^I-containing peptides. In rabbits, neither the ^125^I-relabeled BoNT/A complex nor the free BoNT/A were observed in any distant tissues. The authors concluded that the majority of the neurotoxin remained near the injection sites, either as a complex or in free form.

As discussed in Section 2, it was suggested that BoNT/A inhibits neurotransmitter release from sensory nerve endings by cleaving peripheral SNAP-25 [8,11]. The trigeminal system of a rat formalin-induced model was employed to identify which neurons participate in BoNT/A axonal transport [48,49]. A low dose (3.5 units/kg) of BoNT/A was injected into either the rat whisker pad or sensory trigeminal ganglion (1 unit/kg). Colchicine was injected into the trigeminal ganglion to prevent axonal transport. Both injections of BoNT/A were effective in reducing formalin-induced pain in the rat. Colchicine completely prevented the antinociceptive effects of BoNT/A. After the toxin was injected into the rat whisker pad, BoNT/A-truncated SNAP-25 was immunohistochemically labeled in the medullary dorsal horn of the trigeminal nucleus; it was evident in the spinal trigeminal nucleus three days after the peripheral application of even the low dose. Therefore, in this study, it was suggested that axonal transport is required for the antinociceptive effects of BoNT/A in sensory neurons and that truncated SNAP-25 in central sensory nociceptive nuclei is also associated with these effects [49,50].

There are arguments about whether BoNT are retrogradely transported from the injection site or not. In this review, the mechanism of the antinociceptive action of BoNT/A at the peripheral and/or central level is investigated. Marinelli and colleagues analyzed the expression of cl-SNAP-25 from nerve endings of the hind paw to the spinal cord after applying BoNT/A peripherally [51]. With chronically injured sciatic nerves of mice, immunostained cl-SNAP-25 in the peripheral nerve endings were evaluated along the sciatic nerve including dorsal root ganglia and spinal dorsal horns after the injections. The injections included pure saline, BoNT/A, or combination of BoNT/A with either glial fibrillar acidic protein, complement receptors, or neuronal nuclei. On the all the examined tissues, there were immunostained cl-SNAP-25 from the peripheral nerve endings to the spinal cord; these results support a retrograde transport of BoNT/A. Additional *in vitro* experiments were also done to evaluate whether the proliferative state of Schwann cells would interact with BoNT/A or not. As results, BoNT/A modulated the proliferation of Schwann cells, inhibiting the acetylcholine release. These results support the retrograde transport of the BoNT/A along the nerve.

In the study of Restani *et al.*, the BoNT/A was injected into the adult rat optic tectum, producing SNAP-25 cleavage in retinal neurons, which are distanced from the injection site [42]. The retinal endings showed impaired exocytosis including cleaved, enlarged SNAP-25 and an abnormally high number of synaptic vesicles. The injection produced truncated SNAP-25 in cholinergic amacrine cells. The cholinergic-driven wave activities were decreased on the functional images with calcium indicators, meaning impairments in neurotransmission. These results are consistent with the effects of the retrograde trafficking of BoNT/A.

In another study, it was suggested that intraplantar BoNT/B could have not only acute local effects upon the peripheral afferent nerve but could also transport to the central afferent terminals over time [52]. Each mouse received 1 unit of BoNT/B into its unilateral plantar of the mouse and saline into the contralateral side. After 4 h, 1, 7 and 21 days, they received injections of 1% capsaicin into bilateral plantar surfaces. In the results, the injected side showed local early effect of ipsilateral capsaicin-evoked plasma extravasations. This means local uptake and effect on afferent terminals. The ipsilateral injected side had reduced intraplantar formalin-evoked flinching, capsaicin-evoked plasma extravasation, formalin-evoked dorsal horn substance P release, and formalin-evoked dorsal horn neuronal activation (*c-fas*). It also had reduced ipsilateral dorsal root ganglion VAMP, ipsilateral substance P release otherwise evoked bilaterally by capsaicin, and ipsilateral activation of *c-fas* otherwise evoked bilaterally. Intrathecal substance P-evoked *c-fas* activation is unilaterally blocked by the BoNT/B, suggesting a transsynaptic event. The results of this study suggest that BoNT/B can be taken up by the peripheral terminal and be transported to the ipsilateral dorsal root ganglia (DRG) to cleave VAMP. BoNT/B could act presynaptically. In addition, the observations provided evidence for possible transsynaptic effects of intraplantar BoNT. These results suggest that peripheral BoNT/B can change peripheral and central terminal release from a nociceptor and attenuate downstream nociceptive processing via a presynaptic effect, suggesting a possible postsynaptic effect.

The study of Xu *et al.* [53] did not focus on neuropathic pain, but it did show postsynaptic actions by BoNT/A on the salivary gland. In this study, the authors evaluated the effects of BoNT/A on the apical plasma membrane water channel aquaporin-5 [53]. This study demonstrated that saliva secretion in rat submandibular glands reversibly decreased after the application of BoNT/A. This action was by not only presynaptic SNAP-25 cleavage at the neuroglandular junctions but also the postsynaptic water channel redistribution. The cell surface protein levels of water channels after the applications of BoNT/A reduced without affecting the total protein expression of the water channel, and through immunofluorescence, there was translocation of the water channels from the membrane to the cytoplasm, suggesting the redistribution of the water channels.

## 4. Botulinum Toxin and Inflammation

In peripheral neuropathies, BoNT/A can reduce neurogenic inflammation [54]. In the rat formalin-induced pain model, BoNT/A was injected into the rat paw one week prior to formalin injection [11]. The post-formalin inflammatory peak of pain was reduced for certain doses of BoNT/A. In examining the injection site, both inflammation and the concentration of glutamate were reduced compared with controls. In a capsaicin-induced prostatitis rat model, BoNT inhibited both cyclooxygenase-2 expression [55] and G protein family expression, including Rho guanosine triphosphatase, which activates interleukin-1, a pro-inflammatory cytokine [56]; in this manner, the BoNT injection suppressed capsaicin-induced prostatitis.

It has been proposed that the antinociceptive and anti-inflammatory activities of BoNT have common peripheral mechanisms [57]. The anti-inflammatory effects were thought to affect pain by reducing the release of peripheral neurotransmitters and inflammatory mediators [54]; however, this relationship is still controversial. BoNT/A was injected into an animal model of carrageenan-induced hyperalgesia [46,57] and was effective in reducing mechanical and thermal hyperalgesia, although no improvements in local tissue inflammatory edema or plasma protein extravagation were observed. These results showed a dissociation between the antinociceptive and anti-inflammatory effects of BoNT/A [54]. In a study by Cui *et al.*, the anti-inflammatory effects of BoNT were dose-dependent, whereas its antinociceptive effects were not [11]. Experimental studies in humans have also shown similar results [58]: Pain relief was observed only when capsaicin was injected into a BoNT/A-pretreated area. Neurogenic inflammation, including flare and vasodilatation, was reduced even when capsaicin was injected adjacently into a toxin-pretreated region where pain was not reduced. It has been suggested that the anti-inflammatory effects of BoNT/A on vasodilatation and neurogenic flare do not completely correlate with its antinociceptive effects. The relationship between the anti-inflammatory and antinociceptive effects of BoNT/A is still controversial.

## 5. Clinical Evidence of Botulinum Toxin for Neuropathic Pain

Neuropathic pain is caused by any injury to the peripheral or central nervous system [15]. The results from several double-blind, placebo-controlled studies and increasing evidence from case series and reports have suggested that BoNT/A has antinociceptive effects in neuropathic pain [9], and BoNT/A is expected to be an effective treatment for chronic pain. Francisco *et al.* have suggested that BoNT/A can offer benefits to patients with certain types of neuropathic pain [59]. It is probably effective in relieving postherpetic neuralgia, likely or possibly effective in relieving post-traumatic neuropathic pain, and likely effective in relieving diabetic neuropathy. For allodynia related to postherpetic neuralgia and post-traumatic pain, a neurogenic pain mechanism may be important due to peripheral nerve lesions [60].

There were previous preclinical studies that showed the similar antinociceptive effects of BoNT/A in animal models. In a partial sciatic nerve transection model, BoNT/A reduced neuropathic pain [61]; it also reduced thermal and mechanical hyperalgesia, as well as mechanical and cold allodynia [62]. Additionally, mechanical and cold allodynia were reduced in a spinal nerve ligation model [63]. In a further study, it was also effective in reducing mechanical allodynia in sciatic nerve injury-induced neuropathy [64,65,66]. Recently published clinical studies are reviewed in this section (Table 1).

**Table 1:** Summary of studies on botulinum toxin for neuropathic pain.

References	AAN Class	Study Type (Design)	Number of Patients	Diagnosis	Injection Route/Site/Serotype/Dose	Result
Xiao *et al.* [18] (2010)	I	Randomized, double-blind, placebo-controlled	60	Post-herpetic neuralgia	Subcutaneously/over the area of allodynia/BoNT/A/5 IU per site	VAS reduction and sleep quality improvement; superior to control group
Apalla *et al.* [67] (2013)	I	Randomized, double-blind, placebo-controlled	30	Post-herpetic neuralgia	Subcutaneously/over the affected area in a chessboard manner/BoNT/A/5 IU per point (Total 100 IU)	VAS at least 50% reduction in 13 patients in the intervention group and significant reduction in sleep scored
Ranoux *et al.* [16] (2008)	I	Randomized, double-blind, placebo-controlled	29 (4 post-herpetic)	Post-herpetic neuralgia or post-traumatic/post-surgery neuropathy	Intradermally/into painful area/BoNT/A/20–190 IU	Decreased VAS, burning sensation, allodynic brush sensitivity, a reduced number of pain paroxysms, and improvements in quality of life
Liu *et al.* [68] (2006)	IV	Case report	1	Post-herpetic neuralgia	Subcutaneously/over the all painful area in a fan pattern/BoNT/A/100 IU	VAS reduction from 10 to 1 (lasting for 52 days)
Sotiriou *et al.* [69] (2009)	IV	Case series	3	Post-herpetic neuralgia	Subcutaneously/20 injection in a chessboard pattern/BoNT/A/100 IU	VAS decreased within three days (lasting for 64 days)
Wu *et al.* [70] (2012)	I	Randomized, double-blind, placebo-controlled, parallel design	42 (22 BoNT, 20 placebo)	Trigeminal Neuralgia	Intradermally or submucosally/into trigger zones/BoNT/A/75 IU	Reduction in VAS (>50%) in 68% (BoNT group) *vs.* 15% (placebo group)
Bohluli *et al.* [71] (2011)	IV	Prospective, open, case series	15	Trigeminal Neuralgia	Not specified injection mode/into trigger zones/BoNT/A/50–100 IU	100% improvement in global assessment scale, frequency of pain attacks, and VAS scores
Zúñiga *et al.* [72] (2008)	IV	Prospective, open, case series	12	Trigeminal Neuralgia	Subcutaneously/into trigger zones/BoNT/A/20–50 IU	Reduction in VAS (from 8.8 to 4) and number of paroxysmal attacks in 10 patients (lasting for two months)
Türk *et al.* [73] (2005)	IV	Prospective, open, case series	8	Trigeminal Neuralgia	Two points (depth 1.5–2 cm) around zygomatic arch/BoNT/A/50 IU per point (total 100 units)	Reduction in VAS and the frequency of attacks (100%)
Piovesan *et al.* [74] (2005)	IV	Prospective, open pilot study	13	Trigeminal Neuralgia	Subdermally/painful area in a grid pattern/BoNT/A/3 IU per point (total 6–9 IU)	Reduction in VAS for 60 days (100%: Pain-free (4), more than 50% reduction (9))
Borodic *et al.* [75] (2002)	IV	Prospective, open pilot study	11	Trigeminal Neuralgia	Subdermally or Intradermally/subcutaneous trigger zones (depth 1–3 mm, 10 mm apart)/BoNT/A/total 30–50 IU	Reduction in pain (>50%) in eight patients and frequency (lasting for 2–4 months)
Ngeow and Nair [76] (2001)	IV	Case report	1	Trigeminal Neuralgia	Subcutaneously/two trigger zones over painful area/BoNT/A/100 IU total	Complete pain relief in nasal area and partial at mental region
Yoon *et al.* [77] (2010)	IV	Case report	1	Trigeminal Neuralgia	Subcutaneously/one point in the middle chin/BoNT/A/10 IU	Decreased painful area and pain intensity
Allam *et al.* [78] (2005)	IV	Case report	1	Trigeminal Neuralgia	Subcutaneous/eight points along the area of V1 and V2/BoNT/A/2 IU per point (total 16 IU)	Reduction in pain (lasting for 90 days)
Layeeque *et al.* [79] (2004)	IV	Prospective, non-randomized, placebo-controlled	48 (22 BoNT, 26 control)	Post-surgical neuralgia	Pectoralis major, serratus anterior, and rectus abdominis muscles/BoNT/A/100 IU	Significantly reduced post-surgical pain and facilitated reconstruction with tissue expander
Yuan *et al.* [17] (2009)	II	Randomized, double-blind, placebo-controlled, crossover trial	20	Diabetic neuropathy	Intradermally/into the dorsum of the foot in a grid distribution patterns/BoNT/A/4 IU per site (50 units into each foot)	Significant VAS reduction at one, eight, and 12 weeks after injection (lasting for 12 weeks) and improvement in sleep quality in BoNT group
Ghasemi *et al.* [80] (2014)	I	Randomized, double-blind, placebo-controlled	40	Diabetic neuropathy	Intradermally/in a grid distribution pattern of 12 sites across the dorsum of the affected foot/BoNT/A/100 IU	Reduced NPS scores and DN4 scores and 30% patients pain-free in intervention groups
Kapural *et al.* [81] (2007)	IV	Retrospective, open, case series	6	Occipital Neuralgia	Perineural/occipital nerve block/BoNT/A/50 IU	Reduction of VAS and Pain Disability Index Scores in five of six patients at four weeks
Taylor *et al.* [82] (2008)	IV	Prospective, open, case series	6	Occipital Neuralgia	Perineural/around the occipital nerve/BoNT/A/100 IU	Significant improvement in sharp/shooting pain scores
Breuer *et al.* [83] (2006)	I	Randomized, double-blind, placebo-controlled	20	Carpal tunnel syndrome	Intramuscularly/into three hypothenar muscles/BoNT/B/2500 IU	No difference compared with the placebo group
Tsai *et al.* [84] (2006)	IV	Prospective, open, pilot study	5	Carpal tunnel syndrome	Intracarpally/on each side of the carpal tunnel/BoNT/A/60 IU	Insignificant trend toward pain improvement at three months without change in conduction time by NCS in three patients
Safarpour *et al.* [85] (2010)	III	1. Randomized, double- blind, placebo-controlled study; 2. uncontrolled, unblended, open-label study	14 (8 BoNT/A, 6 control)	CRPS	Intradermally and subcutaneously/into the allodynic area/BoNT/A/5 IU per point (total 40–200 units)	No response to the BoNT in VAS; study terminated prematurely because of injection intolerance
Carroll *et al.* [86] (2009)	III	Randomized, double-blind, placebo-controlled crossover trial	18 (9 crossover study)	CRPS	Lumbar sympathetic block/BoNT/A/Bupivacaine 0.5% + 75 IU of BoNT/A	Longer duration of pain reduction (median 71 days) in BoNT/A group than the control group (median 10 days)
Kharkar *et al.* [87] (2011)	IV	Retrospective, uncontrolled, nblended study	37	CRPS	Intramuscularly/neck or upper limb girdle muscles/BoNT/A/10–20 IU per muscle (total 100 IU)	The 97% patients reported reduction of pain by 43%
Argoff *et al.* [54] (1999)	IV	Prospective, open, case series	11	CRPS	Subcutaneously/into shoulder girdle muscles/BoNT/A/25–50 IU (total 300 IU)	Reduction in VAS, allodynia, and hyperalgesia and improved skin color
Safarpour and Jabbari [88] (2010)	IV	Case series	2	CRPS	Intramuscularly/trigger points in the proximal muscles/BoNT/A/20 IU per site	Reduction in proximal and distal pain of myofascial pain syndrome and CPRS
Wu *et al.* [89] (2012)	III	Prospective, randomized, double-blind pilot study	14	Residual limb pain or phantom limb pain	Intramuscular and cutaneous/subcutaneously/into focal tender points BoNT/A, 50 IU per site (total 250–300 IU)	Reduced residual limb pain, compared with the lidocaine/depomedrol group; not effective for phantom limb pain
Jin *et al.* [90] (2009)	IV	Case series	3	Residual limb pain or phantom limb pain	Electromyography (EMG)-guided injection/into the painful stumps points with strong fasciculation/BoNT/A/500 IU	Significant pain reduction, improved prosthesis tolerance, and reduced pain medication (100%)
Kern *et al.* [91] (2004)	IV	Case report	1	Residual limb pain or phantom limb pain	Into trigger points of the stump/BoNT/A/4 × 25 IU	Almost completely pain-free and reduced pain medication
Uyesugi *et al.* [92] (2010)	IV	Case report	1	Painful keloid	Subcutaneously/throughout the scar in a fan-like distribution BoNT/A, total 100 IU	Reduction in VAS (from 8 to 6) at five weeks and time periods of pain-free increased
Jabbari *et al.* [93] (2003)	IV	Case Report	2	Spinal cord injury	Subcutaneously/into the area of burning pain and allodynia (16 to 20 sites)/BoNT/A, 5 IU per site	Significant improvement in VAS (burning pain and allodynia lasting at least three months)
Han *et al.* [94] (2014)	IV	Case Report	1	Spinal cord injury	Subcutaneously/into 10 most painful sites of each sole/BoNT/A/20 IU per site	Reduction in VAS from 96 mm to 68 mm and decreased intensity of the paroxysmal bursts VAS 23 mm after eight weeks

BoNT/A: botulinum toxin type A; CRPS: complex regional pain syndrome; VAS: visual analog scale.

### 5.1. Post-Herpetic Neuralgia

To examine the direct analgesic effect of BoNT/A, Ranoux *et al.* conducted a randomized, double-blind, placebo-controlled study of 29 patients with focal painful neuropathies [16]. This study included four patients with postherpetic neuralgia; the remainder had post-traumatic/post-operative neuropathies. They received intradermal injections of BoNT/A of between 20 and 190 units into the painful area. After the injections, pain intensity decreased, allodynia to brushing improved, and pain thresholds to cold were reduced [16]. Later, a randomized, placebo-controlled, double-blind study was conducted on 60 patients with PHN [18]. In this class I study, the patients reported significant pain relief after subcutaneous BoTN/A injection compared with injections of placebo and lidocaine. Pain began to subside three to five days after the injection, and the mean visual analog scale (VAS) score was reduced by 4.5 compared with the placebo group (2.9) [18]. Recently, 30 adults with PHN were randomly divided into BoNT/A and placebo groups in a parallel, randomized, double-bind, single-dose, controlled human study by Apalla *et al.* [67]. In this class I study, a total of 100 units of BoNT/A was injected subcutaneously all over each affected area in a chessboard-like pattern. Thirteen patients showed a significant VAS score reduction of at least 50% from the baseline. This significant reduction lasted for a median period of 16 weeks [67]. There have been earlier case reports on the antinociceptive effects of BoNT/A in PHN; Liu *et al.* reported that after the injection of BoNT/A into an allodynia lesion of a PHN patient, the patient’s VAS gradually decreased from a score of 10 to a score of 1; the effect persisted for 52 days [68]. In 2009, Sotiriu *et al.* reported similar results in a case series of three PHN patients [69]. These patients were treated with subcutaneous BoNT/A injections over their affected areas. Their mean VAS score decreased from 8.3 to 2 after two weeks and increased to 4 after three months. In summary, there are two class I studies that show the efficacy of the antinociceptive effects of BoNT/A when used as a treatment for PHN [18,67].

### 5.2. Trigeminal Neuralgia

Wu *et al.* evaluated 42 patients with trigeminal neuralgia in a randomized, double-blind, placebo-controlled study: 22 were treated with BoNT/A, and 20 were treated with a placebo [70]. In this class I study, 75 units of BoNT/A were injected either intradermally or submucosally into the painful areas of each patient in the intervention group. Of the patients administered BoNT/A injections, 68.18% reported more than a 50% reduction in pain intensity on the VAS compared with 15% in the placebo group.

Turk *et al.* conducted a class IV, open-ended study to investigate the effectiveness of administering BoNT/A to patients with trigeminal neuralgia [73]. Eight patients were injected with 100 units of BoNT/A in the area around the zygomatic arch, and all of the patients experienced beneficial effects. In a class IV, open-label study, 13 patients with idiopathic trigeminal neuralgia were transcutaneously injected with BoNT/A at the trigeminal nerve branches [57]. Four patients were pain-free, and nine patients reported more than a 50% reduction in pain intensity as measured by VAS score, which lasted for 60 days. Borodic *et al.* conducted a class IV, open-label pilot study; 44 patients with chronic facial pain were treated with BoNT injections into the painful area, and the diagnoses included temporomandibular joint syndrome, idiopathic trigeminal neuralgia, and post-surgical pain syndrome, among others [75]. Eleven patients with idiopathic trigeminal neuralgia were transcutaneously injected with 25–75 units of BoNT/A. Eight patients reported at least a 50% reduction in pain frequency and intensity, and these beneficial effects lasted for 2–4 months.

In another class IV, open-label study, 20–50 units of BoNT/A were subcutaneously injected into trigger zones in 12 patients with idiopathic trigeminal neuralgia [72]. If mandibular muscles were involved, additional injections were performed in master muscles. Ten patients showed significant improvements in pain based on VAS scores, which lasted for 60 days. At eight weeks post injection, the cumulative mean pain score on the VAS was reduced from 8.83 to 4.08. The number of paroxysmal attacks of pain per 24 h period decreased from 23.4 to 8.8. Similar results were observed in a class IV, open-label study by Bohluli *et al.*, in which 15 patients with trigeminal neuralgia were injected with BoNT/A in their trigger zones [71]. In this study, it was not specified whether the mode of injection was subcutaneous or intradermal. All 15 patients showed improvements in pain frequency and severity after the intervention, which lasted for up to six months. The frequency of attacks per day reduced from 33 to 8; seven patients reported complete eradication of pain after the injection.

There have been several case reports with similar successful results. Both Ngeow and Nair and Allam *et al.* reported cases that exhibited reduced pain after subcutaneous injections of BoNT/A into painful areas [76,78]. In a case report by Yoon *et al.*, BoNT/A was subcutaneously injected into the area surrounding the middle of the chin [77]. The size of the painful area was reduced after one month, and there was a slight pain reduction after two months.

There have been a few case reports and open-label studies, including several class IV studies, that report the beneficial effects of BoNT/A injections for the management of trigeminal neuralgia, but there is only one class I study [70]. BoNT/A injection is likely effective for trigeminal neuralgia, but well-designed, randomized, controlled, double-blind trials are still necessary to investigate the therapeutic efficacy of BoNT/A for trigeminal neuralgia.

### 5.3. Post-Traumatic Neuralgia or Post-Surgical Neuralgia

As mentioned in Section 5.1, Ranoux and colleagues conducted a randomized, double-blind, placebo-controlled study that evaluated eight patients with post-traumatic neuralgia, 17 patients with post-surgical neuralgia, and four patients with postherpetic [16]. In this class I study, BoNT injections were effective at reducing neuropathic pain. The patients were evaluated at baseline and at 4, 12 and 24 weeks after BoNT injection. The patients showed significantly reduced brush allodynia, non-evoked pain, and cold allodynia. There were no changes in their responses to thermal or mechanical pain. These effects began two weeks after the injection and lasted for up to 24 weeks.

In a randomized, placebo-controlled study, 48 patients were injected with 100 units of BoNT/A before mastectomy into the pectoralis major, serratus anterior, and rectus abdominis muscles [79]. In this class IV study, post-surgical pain was significantly reduced, and more pain medication was used by the patients in the placebo group than those in the intervention group. BoNT is likely effective as a treatment for post-traumatic and post-surgical neuralgia, but another well-organized, randomized study is necessary to confirm this conclusion.

### 5.4. Diabetic Neuropathy

Yuan *et al.* concluded that BoNT/A can be an effective treatment for painful diabetic neuropathy based on the results of a randomized, double-blind, placebo-controlled study [17]. In this class II study, there was a significant decrease in the VAS score of a treatment group that included 18 patients with diabetic neuropathy at 1, 4, 8 and 12 weeks (*p* < 0.05). The antinociceptive effects lasted for up to 12 weeks. Additionally, there was improvement in the scores of a Chinese version of the Pittsburgh Sleep Quality Index four weeks after the injection [17]. Furthermore, Chen and Yuan *et al.* conducted another study that suggested the possibility that tactile and mechanical pain reception might be improved by BoNT/A injection in patients with diabetic polyneuropathy [95].

In 2014, a randomized, double-blind, placebo-controlled clinical trial was performed on diabetic patients [95]. In this class I study, the patients were diagnosed with diabetic neuropathy using the Douleur Neuropathique 4 (DN4) questionnaire and nerve conduction studies. They were under 70 years old and presented with neuropathic pain in their feet. Forty patients were randomly assigned into two groups: the BoNT/A and placebo groups. One hundred units of BoNT/A were injected into each patient in the intervention group, and normal saline was injected into the other 20 patients. After the BoNT/A injections, the patients’ neuropathy pain scale (NPS) scores were reduced for all items except cold sensation (*p* < 0.01). Their DN4 scores also decreased, which were related to electric shocks, pins and needles, and burning and brushing (*p* < 0.05). According to the class I and class II studies described above [17,80], BoNT is likely effective as a treatment for diabetic neuropathy.

### 5.5. Occipital Neuralgia

Kapural *et al.* retrospectively analyzed a case series of six patients with occipital neuralgia who received occipital nerve blocks with 50 units of BoNT/A [81]. VAS scores were significantly reduced, and the pain disability index improved in five out of the six patients at four weeks after the BoNT/A injection. The patients reported long-lasting pain relief, which persisted for more than four months in five patients.

In a prospective, open-label, pilot study by Taylor *et al.*, 50 units of BoNT/A were injected inferolaterally into the occipital protuberance in six patients with occipital neuralgia [82]. The patients reported daily ratings of their headache types based on the Visual Analog Pain and Medication Use Diary and recorded their dosages of daily pain medication for 12 weeks after the injection. The authors found that both sharp/shooting and pins/needles types of headache pain significantly improved over the baseline at various time points. Pins/needles pain improved over baseline with statistical significance at 3–6 weeks, whereas sharp/shooting pain improved during weeks 2–3 and 7–12. However, there was no significant improvement in dull/aching pain. None of the patients reported a cessation of their headaches after being injected, and there were no changes in pain medication dosage.

As there have been only two class IV studies examining BoNT/A as a treatment for occipital neuralgia, there is currently insufficient evidence to prove its efficacy in alleviating this condition.

### 5.6. Carpal Tunnel Syndrome

In a randomized, double-blind, placebo-controlled study by Breuer *et al.*, 20 patients were included who had been diagnosed with carpal tunnel syndrome by nerve conduction studies [83]. In this class I study, the patients were randomly assigned and received either BoNT/B or normal saline via intramuscular injection into their hypothenar muscles. The patients were monitored every week for 13 weeks for pain severity based on the VAS score and for opiate usage. The authors found that BoNT/B injection produced no beneficial effects in patients with carpal tunnel syndrome with respect to pain or quality of life compared with the normal saline group.

Tsai *et al.* conducted a prospective, open-label case series that included five females with carpal tunnel syndrome [84]. A total of 30 units of BoNT/A were injected intracarpally on each side of the carpal tunnel. Three out of five patients reported insignificant pain improvement at three months, without electrophysiological changes. According to this class I negative study and the one class IV study with positive results, there is insufficient evidence to prove the efficacy of BoNT as a treatment for carpal tunnel syndrome [83,84]. In both studies, BoNT was delivered differently than in other studies: It was either intramuscularly or intracarpally injected. Thus, the negative results of these studies might be influenced by the mode of administration.

### 5.7. Complex Regional Pain Syndrome

Safarpour and colleagues conducted a randomized, prospective, double-blind, placebo-controlled, open-label extension study; they observed that subcutaneous and intradermal injections of 5 units/site of BoNT/A (40 to 200 units total) into allodynic areas of patients with complex regional pain syndrome (CRPS) were not effective at reducing pain [85]. There were no differences between the intervention and placebo groups. This study was prematurely terminated due to intolerance of the BoNT/A injection.

A randomized, double-blind, placebo-controlled crossover study was conducted to further investigate the antinociceptive effect of BoNT in CRPS patients [86]. Lumbar sympathetic blocks with BoNT/A were performed instead of peripheral application. Pain was relieved for a significantly prolonged period (71 days) in the treatment group, who were administered a combination of local anesthetics and BoTN/A, compared with a group who was administered anesthetic alone (10 days).

Open-label studies have shown pain relief and improvements in skin color and edema in patients with CRPS. In a study by Argoff, 11 patients with CRPS were injected with 300 units of BoNT/A into the muscles in which the patients complained of feeling maximal pain [54]. Each muscle was subcutaneously injected with between 25–50 units of BoNT/A. After 6–12 weeks, all of the patients reported reduced burning sensations and dysesthetic pain in their affected extremities, with normalized skin and decreased edema. Safarpour and Jabbari reported two cases with CRPS who presented with proximal myofascial pain that responded to BoNT/A [88]. These patients had proximal myofascial pain syndromes in the ipsilateral side to the distal painful limb. After BoNT/A injections into trigger points in the proximal muscles, both the proximal pain of myofascial pain syndrome and the distal painful symptoms of CPRS were improved.

Thirty-seven patients with dystonia or spasm in the neck or upper limb girdle muscles were reviewed in a retrospective, unblinded, uncontrolled study [87]. Local pain scores were measured four weeks after intramuscular injections of BoNT/A (10–20 units per muscle) were performed. The mean pain score was reduced by 43% and 97% of the patients reported a reduction in pain.

There have been two class III studies, one with negative and one with positive results, as well as three class IV studies [54,85,86,87,88] showing positive results. The efficacy of BoNT/A has not been proven due to the diverse results from the studies on CRPS patients.

### 5.8. Residual Limb Pain or Phantom Limb Pain

In a prospective, randomized, double-blind pilot study, 14 amputee patients with residual limb pain were intramuscularly, intraneuronally, or subcutaneously injected into painful sites with either 250 to 300 units of BoNT/A or with a combination of lidocaine and depomedrol [89]. The patients were evaluated for residual limb pain and phantom limb pain using the VAS, both at baseline and during each month after the injection for six months. Compared with baseline, both the BoNT/A and lidocaine/depomedrol groups showed immediate improvements in residual limb pain and pain tolerance. These effects lasted for six months in both of the groups, but there were no changes in phantom limb pain.

There was a case report that the antinociceptive effect of BoNT/A was effective in reducing phantom limb pain for over 12 months [91]. BoNT/A was injected into the trigger points of the stump of a lower limb amputee with phantom limb pain. The injections were repeated four times at three-month intervals. The patient’s pain improved, and his dose of intrathecal morphine therapy was reduced to 40% of the initial dose. Intrathecal clonidine and other oral pain medications were completely ceased.

### 5.9. Miscellaneous

In addition to the previously mentioned sections, there have been studies showing the efficacy of using BoNT/A to treat other conditions that include neuropathic pain. There was a case report that neuropathic pain caused by a keloid was successfully treated with BoNT/A injection [92]. After two surgeries, an 80-year-old woman complained of a painful chest wall scar that produced a burning, stinging sensation and allodynia [96]; she was injected with a total of 100 units of BoNT/A, and after five weeks, her pain reduced from 8 to generally 6 and intermittently to 0 out of 10 on the numeric pain scale. There was another case report that BoNT/A was effective at reducing chronic post-thoracotomy pain [79]. A 67-year-old man who had a thoracotomy scar with a predominantly neuropathic component was treated with 100 units of BoNT/A. After two weeks, he reported an improvement in his pain. His VAS score dropped from 6 to 1, and these effects persisted for more than 12 weeks.

There have been several case reports on using BoNT/A to treat neuropathic pain related to spinal cord injury. Jabbari *et al.* reported two patients with burning pain and allodynia from a spinal cord lesion. Both patients had cervical spinal cord injuries and complained of skin sensitivity and burning pain in the dermatome [93]. The burning pain was significantly lessened after BoNT/A was injected into multiple points over each patient’s painful area. Han *et al.* also reported a case with intractable neuropathic pain with a VAS of 96 mm, which was caused by spinal cord injury [94]. The patient reported significant pain relief after 20 units of BoNT/A were injected into the 10 most painful sites on each sole. The patient’s VAS decreased from 96 to 23 mm.

## 6. Administration Routes and Dosage

There are currently no guidelines for the proper mode of administration or dosage of BoNT injection when used as a treatment for neuropathic pain. However, in this review, the above-mentioned injection techniques primarily include either intradermal or subcutaneous injections into painful areas. However, in a study by Breuer *et al.*, BoNT was injected intramuscularly [83]. The results of this study showed no beneficial effects in patients with carpal tunnel syndrome. This mode of administration might have hindered the antinociceptive efficacy of BoNT. In a study by Tsai *et al.*, BoNT was injected intracarpally [84]; the results of this study might also be limited by the route of administration. The mode of administration might play an important role, as BoNT enters into neurons by binding to the synaptic vesicle protein 2 (SV2) receptor [97]. After binding to the SV2 receptor on the membrane, BoNT/A endocytosis proceeds, and BoNT/A is processed in the acidic compartments of small synaptic vesicles.

In the above-reviewed studies, diverse dosages were used; 2.5 to 50 units per point of BoNT were subcutaneously or intrademally injected over 1–40 sites with a total dosage of 6–300 units. Ranoux *et al.* intradermally injected five units using a grid pattern over the painful area, with a maximum dose of 200 units [16]. Liu *et al.* subcutaneously injected BoNT/A over the painful area in a fanning manner, dividing the injections into 20 routes, using five units per route and a maximal dose of 100 units into the area of allodynia [68]. Xiao *et al.* performed subcutaneous injections within 1–2 cm over the area of allodynia, using a maximum of 40 injection sites and depositing five units per site [18]. In a study by Yuan *et al.*, intradermal BoNT/A injections were applied to the foot dorsum in a grid-like distribution pattern, with four units injected per site and 50 units injected per foot [17]. For the treatment of trigeminal neuralgia, Piovesan *et al.* subdermally injected three units per point in a grid-like pattern [74]. Ngeow and Nair performed subcutaneous injections into two trigger zones over the painful area; a total dose of 100 units was used [76]. There was also a report of a lumbar sympathetic block by Carroll *et al.*, who administered 75 units of BoNT/A mixed with bupivacaine [86]. Further studies on what the effective dosages of BoNT/A are must be conducted.

## 7. Potential Adverse Effects

BoNT/A has a high safety profile with few reported irreversible medical adverse effects [98,99]. When a small amount of BoNT/A enters the circulatory system, systemic or regional complications can be produced such as antibody formation and possible immune-related complications. However, to develop antibodies, large amounts of BoNT are required, and amounts of ideal dosages of BoNT for neuropathic pain are not yet known. [98,100,101]

There are trials to measure the safety of BoNT/A for each neuropathic pain disease. For example, Lakhan *et al.* published an article which was a meta-analysis of two studies using BoNT/A for neuropathic pain, especially painful diabetic neuropathy [102]. In their article, there was only one adverse effect, the infection of an injection site, which was not statistically significant. For chronic migraines, according to the Phase III REsearch Evaluating Migraine Prophylaxis Therapy (PREEMPT) clinical study by Aurora *et al.*, there were more adverse events in the BoNT/A group (28.5%) than the placebo group (12.4%) [103]. The most frequently reported side effect in the BoNT/A group included neck pain (4.3%), injection site pain (2.1%), eyelid ptosis (1.9%), and muscular weakness (1.6%). These adverse effects are consistent with the previously reported adverse events from 24-week double-blind and 56-week PREEMPT clinical trials [12,103,104]. In this study, the rate of adverse events decreased progressively as additional injections were done, suggesting cumulative benefits of continued prophylaxis.

## 8. Conclusions

BoNT has become more widely used for the treatment of neuropathic pain. To investigate the antinociceptive effects of BoNT, we assessed levels of evidence from clinical and experimental studies according to the American Academy of Neurology guidelines. According to our assessments, recent studies suggest that BoNT injection is effective as a treatment for postherpetic neuralgia and is likely effective in treating trigeminal neuralgia and post-traumatic neuralgia. BoNT may be effective as a treatment for diabetic neuropathy. Its efficacy has not been proven for the treatment of occipital neuralgia or complex regional pain syndrome.
